# Potential perioperative cardiovascular outcomes in cannabis/cannabinoid users. A call for caution

**DOI:** 10.3389/fcvm.2024.1343549

**Published:** 2024-06-21

**Authors:** Marco Echeverria-Villalobos, Yosira Guevara, Justin Mitchell, David Ryskamp, Joshua Conner, Margo Bush, Luis Periel, Alberto Uribe, Tristan E. Weaver

**Affiliations:** ^1^ Department of Anesthesiology, The Ohio State University Wexner Medical Center, Columbus, OH, United States; ^2^ Department of Anesthesiology, St Elizabeth’s Medical Center, Brighton, MA, United States; ^3^ Department of Anesthesiology & Perioperative Medicine, UCLA Medical Center, Los Angeles, CA, United States; ^4^ University of Toledo, College of Medicine and Life Sciences, Toledo, OH, United States; ^5^ Touro College of Osteopathic Medicine, New York, NW, United States

**Keywords:** cannabis, cannabinoids, cardiovascular effects, perioperative cardiovascular outcomes, postoperative cardiovascular outcomes

## Abstract

**Background:**

Cannabis is one of the most widely used psychoactive substances. Its components act through several pathways, producing a myriad of side effects, of which cardiovascular events are the most life-threatening. However, only a limited number of studies address cannabis's perioperative impact on patients during noncardiac surgery.

**Methods:**

Studies were identified by searching the PubMed, Medline, EMBASE, and Google Scholar databases using relevant keyword combinations pertinent to the topic.

**Results:**

Current evidence shows that cannabis use may cause several cardiovascular events, including abnormalities in cardiac rhythm, myocardial infarction, heart failure, and cerebrovascular events. Additionally, cannabis interacts with anticoagulants and antiplatelet agents, decreasing their efficacy. Finally, the interplay of cannabis with inhalational and intravenous anesthetic agents may lead to adverse perioperative cardiovascular outcomes.

**Conclusions:**

The use of cannabis can trigger cardiovascular events that may depend on factors such as the duration of consumption, the route of administration of the drug, and the dose consumed, which places these patients at risk of drug-drug interactions with anesthetic agents. However, large prospective randomized clinical trials are needed to further elucidate gaps in the body of knowledge regarding which patient population has a greater risk of perioperative complications after cannabis consumption.

## 1 Introduction

Cannabis, an ancient plant that was originally used as a medicinal herb but also as a recreational drug, has been used since the third millennium BC and is known to have many therapeutic effects (1). It was originally cultivated in central Asia but is now grown all over the world (1). However, until recently, the cardinal use of cannabis has been as a recreational drug for pleasurable psychological effects, mainly from delta-9-tetrahydrocannabinol (THC), the principal psychoactive component of the cannabis plant (1, 2). Nevertheless, the wide therapeutic potential of cannabis has led to much usage and research, although additional studies to determine the safety and efficacy of natural cannabis and synthetic cannabinoids are still needed. Cannabis species possess multiple functions and have been utilized as prospective relief agents for epilepsy, neurodegenerative diseases, posttraumatic stress disorder, rheumatic diseases, chemotherapy-induced nausea and vomiting (CINV), and chronic pain (3–10).

Although caffeine is the most commonly used and accessible drug among the general population, cannabis is the most frequently used of all psychoactive substances, following alcohol and tobacco (11). Cannabis use has been legalized in 24 states (including Guam, the Northern Mariana Islands, and DC). In comparison, 38 states and DCs allow the medical use of cannabis products, and 10 states in the U.S. (and the U.S. Virgin Islands) have decriminalized its use (12), which has increased the availability and diversity of cannabis-containing products. Moreover, in the last decade, the average concentration of THC cannabis has sharply increased from 2% to 3% in the 1970s to more than 20% in 2017, largely due to the growth of more potent strains (13, 14). The “shatter” or “butane hash oil”, also known as “the crack of marijuana”, hits the streets very rapidly in the U.S. It is a cannabis oil concentrate produced by butane solvent that allows the extraction of a cannabis product with a very high concentration (90%–95%) of delta-9-tetrahydrocannabinol (THC) (15).

The most well-known cannabinoids derived from the cannabis plant are delta-9-tetrahydrocannabinol (THC), which has potent psychotropic properties; cannabinol (CBN), which has weak psychoactive effects; and cannabidiol (CBD), which is known for its non-psychotropic properties (16). These chemicals have complicated interactions with endogenous receptors in the endocannabinoid system and the autonomic nervous system and with additional pathways, which can cause unwanted and dangerous physiological responses (11). Synthetic cannabinoids are 10–200 times more potent than naturally grown cannabis, and their increasing availability has correlated with an upsurge in serious adverse events, including renal toxicity, respiratory depression, hyperemesis syndrome, cardiovascular events, and effects on brain function (11, 2). The recreational use of cannabis and synthetic cannabinoids is more frequent in the young population, while medicinal cannabis is more common in the middle-aged population and older individuals (17).

Cardiovascular events are among the risks of cannabis and cannabinoid consumption, as both natural and synthetic cannabinoids induce changes in the cardiovascular system of humans and animals (18). Due to the cardiovascular effects of cannabis, surgeons, and anesthesiologists will be required to take precautionary measures when cannabis users require surgery. The most common route of consumption of cannabis for recreational usage is by inhalation (smoking and vaping), but the oral route in the form of edibles such as “grass” brownies, marijuana tea, and marijuana tincture is increasing (19). Perioperative cardiovascular events resulting from the consumption of cannabis products may differ according to the dose and time of consumption, time of last exposure, potency of the product, composition of the products, route of administration, and concomitant use of other drugs or medications.

There is little data regarding the impact of cannabis use combined with anesthetic drugs and its correlation with an increased risk of perioperative complications in patients enduring noncardiac surgery (20). Consequently, we conducted a literature review to first identify the possible mechanisms underlying the harmful cardiovascular effects of cannabinoids; second, to review the pharmacological interactions of cannabinoids with commonly prescribed drugs, as well as anesthetic agents, in patients with heart disease; and third, to highlight the perioperative cardiovascular consequences associated with cannabis and cannabinoid consumption based on the most recently published evidence.

## 2 Materials and methods

**Figure 1:**
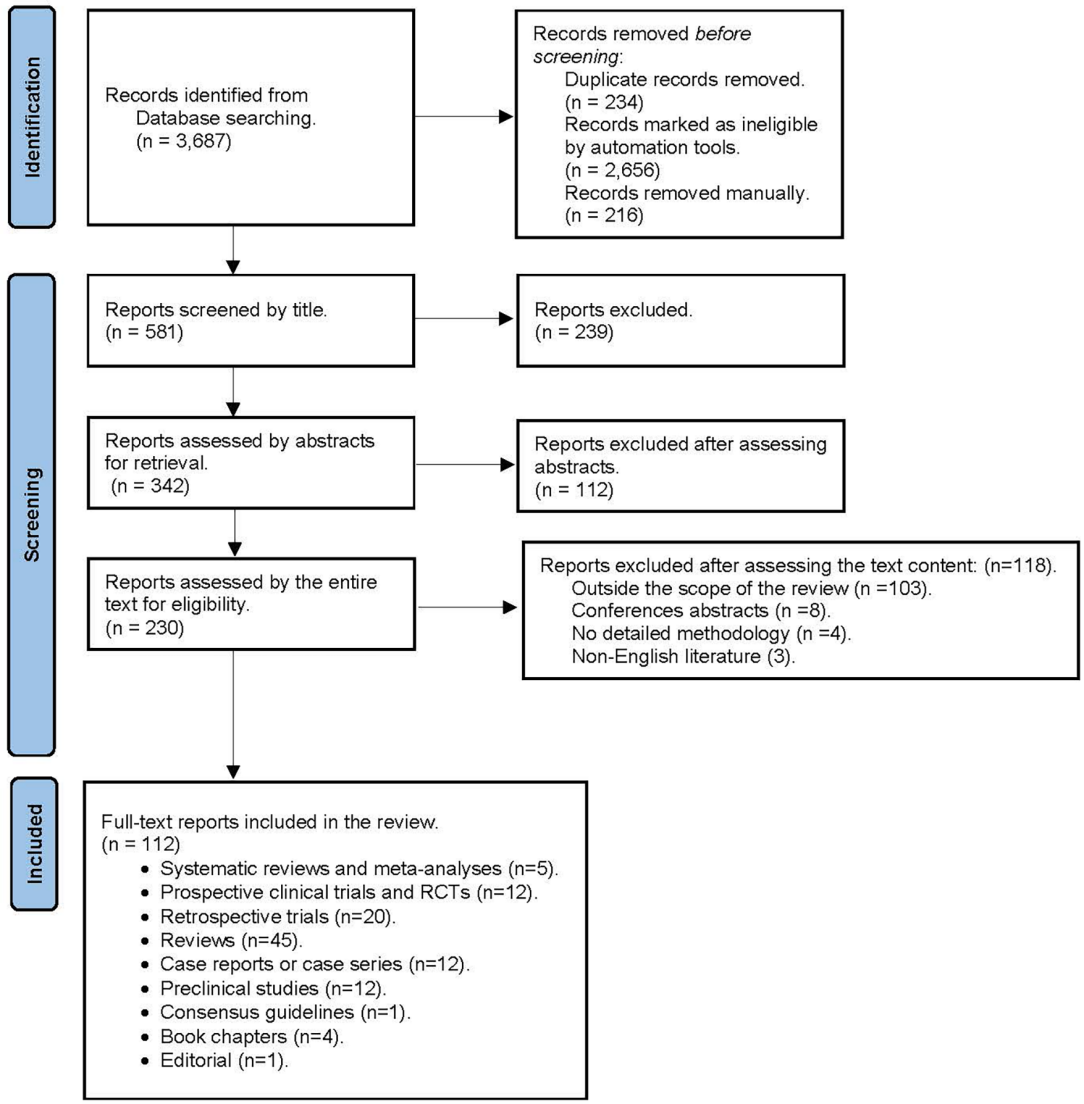
PRISMA diagramn.

A broad search of the current literature on the perioperative cardiovascular effects of cannabis and cannabinoids was performed via PubMed, Medline, EMBASE, Google Scholar, and Web of Science to identify articles published in the English language between January 2015 and June 2022. We also searched for cardiovascular effects and perioperative cardiovascular outcomes in cannabis or cannabinoid users undergoing surgery using the following terms to search for the listed keywords: “(“cannabis”/exp OR cannabis OR “cannabinoids”/exp OR cannabinoids) AND {“perioperative cardiovascular outcomes” OR [perioperative AND (“cardiovascular”/exp OR cardiovascular) AND (“outcomes”/exp OR outcomes)]} OR“postoperative cardiovascular outcomes” OR [postoperative AND (“cardiovascular”/exp OR cardiovascular) AND (“outcomes”/exp OR outcomes)]”. Manuscripts disclosing no methodology or with no full-text availability were excluded from our narrative review (Figure 1 PRISMA diagram). The authors also searched the reference lists of the included studies.

## 3 Discussion

The endocannabinoid system is a complex cell signaling network composed of cell receptors that respond to endogenously produced endocannabinoid ligands and exogenous natural and synthetic agonists and the enzymes responsible for their synthesis and degradation (21). Cannabinoid receptors (CB-Rs) are G protein-coupled receptors (GPCRs) that are extensively distributed in the central and peripheral nervous systems, as well as in other organs and systems. CB1 receptors (CB1-Rs) are predominantly expressed in the brain cortex, hippocampus, dentate gyrus, striatum, amygdala, basal ganglia outflow, nucleus accumbens, thalamus, hypothalamus, periaqueductal gray, brainstem, and cerebellum (22). In the central nervous system (CNS), CB1_Rs are located primarily on neurons [e γ-aminobutyric acid (GABA)ergic, CCK-positive interneurons] and axons (presynaptic terminals), where they modulate neurotransmission (21). CB1_Rs are also found in the peripheral nerves, spinal cord, and enteric nervous system (ENS) (22). CB2_Rs are expressed mainly in leucocytes, the spleen, the thymus, tonsils, and mast cells and modulate cytokine release and the immune response of T cells (23). However, some studies in animals and human tissue have revealed the presence of CB2_Rs in glial cells and neurons, as they function as modulators of differentiation and proliferation of neuronal and nonneuronal cells (24). A list of the exogenous and endogenous ligands, agonists, and antagonists is provided in Table 1.

**Table 1:** Cannabinoid receptor agonists and antagonists.

CB Receptors	Exogenous agonists	Endogenous agonists	Antagonists or inverse agonists
CB1_R/CB2_R	**Δ^9^THC** (phytocannabinoid) • Partial agonist • Efficacy CB1_R>CB2_R **Cannabidiol (CBD)** • Partial agonist (weak affinity) • Highest potency to TRPV1-R **HU 210** (synthetic) • High CB1-R/CB2_R affinity • Full CB1-R/CB2_R agonist **CP55,940** (synthetic) • High CB1-R/CB2_R affinity • High CB-R1/CB2_R agonist **WIN 55,212-2** (synthetic) • High CB1-R/CB2_R affinity • FullCB1-R/CB2_R agonist **Synthetic cannabinoids are more used in humans (K2, Spice))** • High CB1_R/CB2_R affinity • Full CB1_R/CB2_R agonist • **JWH-018** • **IWH-019** • **JWH-073** • **JWH-250** • **AM-2201**	**Anandamide** • Moderate affinity (CB1_R>CB2_R) • Partial agonist (CB1_R>CB2_R) **2- Arachidonoglycerol** • Moderate affinity CB1_R/CB2_R • High CB1_R/CB2_R efficacy	• **Rimonabant** • **Surinabant** • **Ipinabant** • **AM-251**
CB2-R			• **SR 144528** • **AM680**
TRPV1-R		Anandamide (AEA)	** **
TRPA1-R	Cannabinol (CBN)	** **	** **

CB1_R, cannabinoid receptor type-1; CB2_R, cannabinoid receptor type-2; Δ9-THC, delta-9-tetrahydrocannabinol; TRPV1, transient receptor potential V1 channel; TRPA1, transient receptor potential ankyrin 1.

Unfortunately, there is a paucity of well-designed prospective randomized trials with large sample sizes to assess the safety of preoperative use of cannabis and cannabinoids more accurately. In the following sections of this review, we will present evidence of the potential cardiovascular events triggered in the perioperative period by the use of cannabis and cannabinoids.

### 3.1 Cardiovascular effects of cannabis and cannabinoids

The first scientific publications on the use of cannabis suggested that the use of THC caused adverse cardiovascular events only in individuals with cardiac comorbidities and that, on the contrary, its use was safe in healthy people (25). Since then, several adverse cardiovascular events have been reported related to the recreational use of these substances, to the point that cannabis use has been included in the list of risk factors for myocardial infarction (MI) in young individuals without previous cardiac disease (26). The reported perioperative cardiovascular effects associated with the consumption of cannabis and synthetic cannabinoids are related not only to the consumed dose and route of administration of these drugs but also to their pharmacological interactions with cardiovascular medications, platelet antiaggregants, and anesthetic drugs. The most reported cardiovascular effects of cannabinoids include cardiac arrhythmias, coronary vasospasm, myocardial infarction, cerebrovascular ischemic events, stress cardiomyopathy, and sudden death (Table 2).

**Table 2:** Cardiovascular disorders associated with cannabis/cannabinoid use.

System Outcomes	CB-Receptor	Duration of Use	Physiologic effects
Cardiovascular	CB1-R	Newly users/naïve	• Initial β-adrenergic effect + parasympathetic inhibition: ↑HR, ↑Contractility, ↑SBP, AFib, malignant arrhythmias (VTach, VFib) (27–30) • In young consumers: ↑VO2, ↓coronary blood flow, ↑COHb, coronary thrombosis → AMI (31–33)
		Chronic use	• Parasympathetic stimulation + baroreflex inhibition: ↓HR, orthostatic hypotension with no compensatory response, cardiac (24, 29, 30) • Cannabis smoking reduces angina threshold in patients with chronic stable angina (34) • Coronary vasospasm in patients with CAD → AMI (61, 63–65, 82–85, 84) • Coronary thrombosis → AMI (35, 36) • Accelerated atherosclerosis (37, 38) • Hyperadrenergic state → LV systolic dysfunction→ Heart Failure (88–94)
		Synthetic cannabinoids (K2, Spice)	• Prolonged QTc → Cardiac arrest (39, 40) • Nonischemic LV dysfunction (29, 41) • Myocardial infarction (30, 42)
Cerebrovascular	CB1-R	Chronic/heavy user/SCB/K2	• Ischemic stroke (43–47) • Cerebral vasospasm (50% anterior cerebral circulation)(48, 49) • Cerebral thrombosis (K2, Spice) (44) • Transient ischemic attacks (13, 45) • Aneurysmal cerebral hemorrhage with delayed cerebral ischemia (50)
Coagulation	CB1-R	Chronic/Heavy users	• THC, CBD, CBN inhibit CYP2C9 enzyme system →↑ plasmatic levels of warfarin104,^,^(103, 105–107) • CBD inhibits CYP2C19 →↓ plasmatic levels of clopidogrel (51, 52). • CBD interferes with DOAC elimination → ↑ levels of Factor Xa inhibitors (rivaroxaban, apixaban, edoxaban) (48, 11, 53)

CB-Rs, cannabinoid receptor; CB1-Rs, cannabinoid receptor type 1; CBF, cerebral blood flow; HR, heart rate; LV, left ventricle; CO, cardiac output; SBP, systolic blood pressure; VPCs, ventricular premature contractions; AFib, atrial fibrillation; VTach, ventricular tachycardia; VFib, ventricular fibrillation; THC, tetrahydrocannabinol; LV, left ventricle; MI, myocardial infarction; AMI, acute myocardial infarction; MVO2, myocardial oxygen consumption; COHb, carboxyhemoglobin; CGRP, calcitonin gene-related peptide; VR, vanilloid receptor; CBF, cerebral blood flow; SCB, synthetic cannabinoids.

Below, we describe the cardiovascular effects elicited by the main cannabinoid components of the cannabis plant, as well as the most commonly used synthetic cannabinoids.

### 3.2 Phytocannabinoids

#### 3.2.1 Delta-9 tetrahydrocannabinol (Δ9-THC)

Δ^9^-THC is a partial agonist of CB1_R and CB2_R. Its CB1_R partial agonism is responsible for its psychoactive effects due to the extensive-expression of these cannabinoid receptors in the brain (54). THC can increase the expression and activity of PPARα, improving endothelial cell function and protecting them against ischemia and high intraventricular pressure (27, 28). The most reported cardiovascular outcomes of THC in humans are related to enhanced sympathetic activity or blunted parasympathetic tone, with subsequent increases in heart rate, contractility myocardial oxygen consumption, and peripheral vasoconstriction (55, 38).

#### 3.2.2 Cannabidiol (CBD)

The phytocannabinoid CBD does not exhibit psychoactive effects; it is a CB1_R negative allosteric modulator and CB2_R partial agonist. CBD has cardioprotective effects through its ability to modulate the balance of intracellular Ca^2+^ and myocyte contraction, especially under ischemic conditions or in patients with arrhythmias (56, 57). Through PPARγ activation, CBD increases the concentration of nitric oxide (NO) with subsequent systemic and pulmonary vasodilation (58). CBD blockade of the GPR55 receptor contributes to regulating myocardial homeostasis during myocardial ischemia (59, 60).

#### 3.2.3 Cannabinol (CBN)

Cannabinol is a phytocannabinoid produced by the oxidation of THC and is found at very low levels in plants. CBN is also a partial agonist of CB1_R and CB2_R, which can produce mild psychoactive reactions in large doses (61). Preclinical studies in rats have reported a reduced heart rate in response to CBN; however, this effect has not been found in humans (62, 63). The potential cardioprotective effects of CBNs could be related to agonistic interactions with CB2_Rs, TRPV1, and TRPV2 (64, 65).

#### 3.2.4 Cannabigerol (CBG)

It is a phytocannabinoid compound that is also a substrate for the synthesis of Δ9-THC and cannabidiol CBD. CBG shows some similarities with CBD. CBG is a partial agonist for CB1_R and CB2_R; however, it also has agonist effects on other receptors, such as TRPA1, TRPV1, TRPV2, TRPV3, TRPV4, PPARγ, and PPARα (66). CBG also has agonist effects on the α2-adrenoceptor endothelial receptor and antagonistic effects on presynaptic 5H T1A receptors, favoring peripheral vasodilation and lowering blood pressure (67, 68). A study by Willoughby et al. showed that CBG inhibits platelet aggregation (43).

### 3.3 Synthetic cannabinoids

Synthetic cannabinoids are full CB1_Rs and CB2_Rs agonists. They were first produced in the 1970s as a new therapeutic alternative to treat cancer pain (44). These compounds are functionally similar to Δ9-tetrahydrocannabinol (THC) but bind more than 100 times more tightly to the CB1 receptor than to THC, and have long half-lives, therefore its psychotropic effects are more prolonged. Although they are labeled as “not for human consumption”, it has been reported a progressive increase in the consumption of synthetic cannabinoids, especially in the form of “herbal smoking blends” or “herbal incense “ under trade names or brands like K2 or “Spice”, particularly in high schoolers and college students (39). Synthetic cannabinoids are generally sold blended with finely cut plants with no psychotropic effects to give the impression of being a natural product, wrapped in brightly colored metal-foil packages. The most used synthetic cannabinoids or K2 are JWH-018, AM-2201, JWH-019, JWH-073, and JWH-250. Several cardiovascular events have been reported, the most common being serious ischemic stroke of thrombotic etiology (40, 41); cardiac arrest, probably resulting from abnormalities in cardiac conduction (prolonged QTc interval) (29, 30); nonischemic left ventricular dysfunction (42, 69); and myocardial infarction (70, 71). Importantly, synthetic cannabinoids are not detected by the common cannabis urine test. Most cannabinoid receptor antagonists are synthetic cannabinoids.

### 3.4 Medical cannabinoids

The term medical marijuana is used to design any cannabis product regardless of processing method, dose, or route of administration. The increasing knowledge about the molecular pharmacology of the endocannabinoid system has led to an exponential increase in the use of cannabinoids as a therapeutic agent in the last 20 years, especially in the form of natural and synthetic analogs of THC, and natural CBD (72, 73).

Nevertheless, the body of evidence in the literature on serious cardiovascular outcomes among medical cannabis users with potential or established heart disease is scant and weak. Most publications refer to observational studies, case reports, and case series in patients receiving natural THC or synthetic THC analogs due to their direct cardiovascular effects as well as their interaction with some of the medications they receive for their heart condition (74–77). A recent meta-analysis conducted by Watanabe et al, which included 47 studies (2,800 patients), to assess the existing evidence on cardiovascular events associated with medical cannabinoid consumption revealed that there was an increasing risk of orthostatic hypotension (RR 3.55), hypotension (RR 3.55), and tachycardia (RR 1.94), while no serious adverse outcomes were reported. Also, they reported that the oral route of administration posed a higher risk for hypotension compared with those administered by another route (oromucosal spray, anal). Another important finding of this meta-analysis is that compounds based on THC alone tend to produce adverse cardiovascular effects (hypotension, orthostatic hypotension, syncope) than those containing a balanced combination of THC and CBD (76). Below are the cannabis products authorized for medicinal use by the FDA and the most important hemodynamic effects that have been reported.

#### 3.4.1 Dronabinol (marinol, syndros)

Is a synthetic formulation of 9ΔTHC for the treatment of anorexia in patients with AIDS, as well as for nausea and vomiting associated with chemotherapy. Dronabinol-induced sympathomimetic activity may result in tachycardia, but subjects can also experience orthostatic hypotension and/or syncope upon abrupt standing (31, 76).

#### 3.4.2 Nabilone (cesamet)

A synthetic analog of THC with partial agonistic actions on CB1 and CB2 receptors, is used for treating chemotherapy-associated nausea and vomiting. Nabilone has been shown to produce a dose-dependent increase in heart rate and a decrease in systolic blood pressure (31).

#### 3.4.3 Cannabidiol (epidiolex)

A purified form of CBD, is currently used in the management of epilepsy resistant to standard treatment, such as Lennox-Gastaut syndrome and Dravet syndrome in patients 2 years of age and older, and for patients ≥1-year-old with Tuberous Sclerosis Complex (TS) (78–32). Although there are no reports of serious cardiovascular adverse events in patients medicated with Epidiolex, cannabidiol has the potential to decrease resting systolic blood pressure (33, 37).

#### 3.4.4 Nabiximols (sativex)

A formulation of ethanol cannabis extract with a 1:1 concentration of THC/CBD, used as an oromucosal spray in the treatment of multiple sclerosis spasticity and chronic pain. It has been reported the occurrence of postural and orthostatic hypotension, especially with the initial doses (81).

A systematic review recently published by Jouanjus et al. showed strong evidence linking the use of cannabis with a high risk of cardiovascular events, particularly cerebrovascular ischemic stroke, in young males (31 years and 82% more than females) (74). A retrospective cohort study from a nationwide dataset that included 379,843 patients revealed that the rate of admission for acute myocardial infarction (AMI) in young cannabis users increased by 32%, predominantly in males (79.1%), with an increasing trend in in-hospital mortality of 60% over 4 years (82). A recent cross-sectional study of pooled data from the Behavioral Risk Factor Surveillance System showed that frequent marijuana use was associated with significantly greater probabilities of stroke (81%) and myocardial infarction or coronary artery disease (88%) (83).

Myocardial infarction (MI) and cerebrovascular events can occur in otherwise healthy individuals after heavy cannabis smoking (26, 34, 82). A cohort study conducted by Ladha et al. revealed that in 4,610 respondents (out of 33,173 young adults) who reported recent marijuana use, MI was more frequent among recent cannabis users (1.3%) than among nonusers (0.8%) (34). Likewise, there is evidence that in patients with preexisting coronary artery disease, the use of cannabis may increase the chances of MI recurrence (84). A retrospective study performed by Desai et al. using records from the National Inpatient Sample (NIS) reported a higher rate of MI admissions in cannabis users than in noncannabis users (67% vs. 41%), revealing the same trend in admissions for arrhythmias (5% vs. 2.8%), with the aggravating factor that cannabis users were younger and had a lower incidence of cardiovascular comorbidities (84).

Cannabinoids hamper nuclear receptors in hepatic microsomes, leading to changes in the activity of the P450 enzymatic system (CPY) and the UDP-glucuronosyltransferase (UGT) family of enzymes. Nasrin et al. recently demonstrated the inhibitory effect of major cannabinoids and their active metabolites on several CPY enzymes, especially CYP2B6, CYP2C9, CYP2D6, and CYP3A4, as well as on several UDP-glucuronosyltransferase families of enzymes (UGT1A6, UGT1A9, UGT2B4, and UGT2B7) (35, 85). The inhibition of these important enzymatic systems for metabolism and clearance plays a vital role in the pharmacologic interactions of cannabinoids with cardiac drugs, anticoagulants, platelet antiaggregants, hypnotics, and inhalational anesthetics.

### 3.5 Proposed mechanisms for cardiovascular outcomes in cannabinoid users

Next, we will examine the most important mechanisms proposed to explain the cardiovascular effects related to the use of cannabis and cannabinoids.

#### 3.5.1 Cannabinoids and arrhythmias

In the 1970s, Beaconsfield et al. described for the first time that marijuana smoking in healthy volunteers who were non-cannabis users was associated with increased heart rate and electrocardiographic changes such as a decreased amplitude of the P wave—which relates to atrial alterations—and inversion of the T wave (36). Since then, a broad range of abnormal electrocardiographic patterns, such as atrial fibrillation, flutter, atrioventricular block, ventricular tachycardia, and Brugada phenocopy, have been reported after marijuana use (86). Tachycardia is the most relevant cardiovascular effect of THC and induces complex hemodynamic consequences, such as a decrease in stroke volume and an imbalance between myocardial oxygen supply and demand.

The mechanisms underlying arrhythmias after cannabis use are not entirely known, but researchers have proposed different theories through animal model studies (86). Thus, cannabis-induced arrhythmias might be caused by concurrent mechanisms rather than solely by sources. Next, we address the potential mechanisms involved in the development of arrhythmias as a consequence of cannabis use.

##### 3.5.1.1 Autonomic dysregulation

Cannabinoids can cause bradyarrhythmia or tachyarrhythmia in a dose-dependent manner and determine the balance of sympathetic-parasympathetic nervous system activation. Low to moderate doses stimulate the sympathetic system and the release of norepinephrine, increasing sinus automaticity and facilitating conduction through the sinoatrial and atrioventricular nodes, favoring tachyarrhythmia. In contrast, high doses of cannabinoids stimulate the parasympathetic system and cause bradyarrhythmia (87). However, a role for CB1_R activation in the brain has recently been suggested. THC can activate inhibitory presynaptic CB1_Rs located in neurons of the autonomous nervous system (ANS) in animals and humans. In animals, THC elicits bradycardia most likely through the inhibitory action of presynaptic CB1_Rs, whereas in humans, stimulation of central CB1_Rs can offset the inhibition of presynaptic CB1-Rs in sympathetic neurons (26). The reported dissimilar cardiac effects of THC in animals and humans can be explained by the differences in parasympathetic-sympathetic tone predominance between species (88).

##### 3.5.1.2 Dysfunction of voltage and ligand-gated ion channels

Cannabinoids interact directly with Na ^+ ^- and L-type Ca^2^+ channels in ventricular myocytes (89). In a study with rats, the authors found that the endogenous ligand anandamide (AEA) caused a reduction in the maximal amplitudes of Na ^+ ^- and Ca^2+^ currents and, subsequently, the amplitude of the action potential (89). Barana et al. demonstrated that endocannabinoids and cannabinoid analogs block human cardiac potassium (Kv1.5) channels, which are crucial for modulating the duration of the atrial action potential (90).

#### 3.5.2 Cannabinoids and myocardial infection

Data have suggested that marijuana smoke triggers myocardial ischemia, which can range from temporary ischemia to ST-elevation myocardial infarction (STEMI). In addition to marijuana, other synthetic cannabinoid preparations, including MI, are associated with adverse cardiovascular effects (91, 92). Apart from the prototypical mechanisms of MI, such as intravascular thrombus formation and coronary constriction with altered coronary flow, other mechanisms, including autonomic overstimulation, toxic smoke compounds, platelet dysfunction, accelerated atherosclerosis, and increased ROS formation, have been proposed for cannabis-induced MI (38, 93).

##### 3.5.2.1 Sympathetic stimulation

Any modality of cannabis consumption increases heart rate and blood pressure via stimulation of the sympathetic nervous system (36, 94). THC-induced tachycardia is the most relevant cardiovascular effect of cannabis compounds in humans (95). Tachycardia leads to more complex hemodynamic consequences, such as a decrease in stroke volume, which, paired with a reduction in diastolic coronary flow and high levels of carboxyhemoglobin from inhaling combustion products, causes a mismatch between myocardial oxygen supply and demand (26, 96). Several studies in humans have revealed that inhaled THC is accompanied by a rise in blood pressure that could be related to an elevated heart rate, while a study conducted by Crawford and Merrit reported moderate hypotension and tachycardia with cannabis smoking.

##### 3.5.2.2 Myocardial endothelial dysfunction

The activation of CB1-Rs expressed on human endothelial cells has a pro-inflammatory effect, causing oxidative stress through the accumulation of intracellular ROS and the stimulation of mitogen-activated protein kinase (MAPK), ultimately leading to endothelial cell injury and the migration of vascular muscle smooth cells, which stimulates endothelial cell-mediated coronary vasoconstriction and elicits a vasodilatory response (38, 86, 97).

However, activation of CB2_R has the opposite effect, inhibiting endothelial cell adhesion and vascular smooth muscle cell migration (98). Aronow and Cassidy performed a study to evaluate the effect of marijuana on cardiovascular function and exercise-induced angina in 10 men with underlying severe coronary disease, none of whom had previously consumed marijuana. The participants reported a decreased exercise duration (48%) until the onset of angina pectoris after smoking one marijuana cigarette compared to after smoking one placebo cigarette (8.6%) (96). Another study in animal models showed that even only one minute of secondhand marijuana smoke exposure could impair vascular endothelial function; rat blood vessels took at least 90 min to recover function, whereas when breathing secondhand tobacco smoke, their vascular function recovered within 30 min (99). Angiographic studies in patients presenting with ischemic angina or MI after cannabis consumption have corroborated the findings of preclinical studies showing prolonged impairment in arterial flow-mediated dilation (FMD) after short marijuana exposure, correlating the state of low coronary flow with the appearance of ventricular tachycardia (44, 99). In contrast to those of THC, the results of a preclinical study in a rat model showed that CBD has significant cardioprotective effects by reducing the postmyocardial infarction inflammatory response and infarct size (100). Cannabidiol-mediated cardioprotection is also related to its ability to modulate the enzymatic activity of glutathione reductase and glutathione peroxidase, which inhibit the production of ROS, thus protecting myocardial endothelial cells from peroxide-related damage (101). Other *in vitro*, ex vivo, and *in vitro* studies have demonstrated the anti-remodeling, ventricular function restoring, and vasorelaxant effects of CBD (33, 102–105).

##### 3.5.2.3 Platelet dysfunction and potential for bleeding

Several authors have described the association between cannabinoids and coronary thrombosis as a potential cause of myocardial infarction even in patients without previous cardiac comorbidities (106). CB1 and CB2 receptors are present on the platelet surface, and THC exposure can cause changes in membrane phospholipids and increase the surface expression of glycoprotein IIb/IIIa and P selectin, which might contribute to a receptor-dependent pathway of THC-induced platelet activation (107). Conversely, in an animal study, De Angelis et al. reported that the endocannabinoid AEA decreases platelet aggregation by reducing α-granule secretion and decreasing glycoprotein IIB/Ia activation (108). One study *in which rhesus macaques* consumed daily THC edibles also showed a significant reduction in platelet aggregation (109). Despite contradictory evidence regarding the pro- or anticoagulation effects of cannabinoids, there is evidence supporting the modulatory effect of cannabinoids on platelet function and the coagulation system, and multiple factors, such as dosing, route, frequency, and individual sensitivity, might play a role (110). On the other hand, several authors have demonstrated that the endocannabinoid ligands AEA and 2-AG can induce platelet activation and promote aggregation in human platelets (111–113).

##### 3.5.2.4 Vasospasm and altered coronary flow

Case reports of patients with myocardial infarction after marijuana use and a normal angiogram without evidence of underlying atherosclerosis suggest altered coronary flow due to vasospasm as the possible cause of myocardial infarction (114–116). This theory could be supported by the findings of one study in rats, which showed that intra-arterial administration of THC produced increased perfusion pressure, suggesting vasoconstriction (117). Furthermore, cannabis use is believed to cause a mismatch of blood flow supply and demand in the myocardium, but its role in the development of ischemic cardiomyopathy requires additional study (51, 55).

##### 3.5.2.5 Accelerated coronary atherogenesis

CBR1 receptors found within the vascular endothelium are linked to atherogenesis. ROS plays a critical role in atherosclerosis by causing LDL oxidation, apoptosis, and plaque rupture (100). Human studies have shown that CB1_Rs are expressed on macrophages within coronary atheroma (118). The current evidence from preclinical studies is insufficient to conclude that platelet aggregation promoted by THC and synthetic cannabinoids is a leading cause of intracoronary thrombus formation and MI. However, atheromatous plaque formation and rupture secondary to CB1_R activation could be predisposing factors for thrombus formation.

#### 3.5.3 Cannabis and heart failure

Although an association has been identified between marijuana consumption and the onset or worsening of heart failure (HF), additional research studies are needed. Kalla et al. demonstrated via retrospective analysis of a national inpatient database that marijuana usage was an independent predictor of heart failure (52). Although the incidence of hypertension appears to be much greater in marijuana users than in nonusers, the direct effect of marijuana on hypertensive heart failure has not been well established (52).

The inconsistency observed in the data linking marijuana to the development of heart failure may be due to the diverse cardiovascular effects of cannabinoids. As mentioned above, THC increases ROS production, while cannabidiol inhibits ROS production (119). Therefore, the contribution of cannabis products to the development and progression of heart failure is related to ROS production inside the myocardium.

Cannabis has been implicated in left ventricular systolic dysfunction and myocardial stunning, which are the hallmarks of stress cardiomyopathy and Takotsubo cardiomyopathy, respectively (120). Stress cardiomyopathy is a clinical syndrome characterized by acute left ventricular (LV) systolic and diastolic dysfunction secondary to a hyperadrenergic state (48). The proposed deleterious remodeling of the heart muscle in response to a cannabis-induced catecholamine surge is believed to be mediated by CB1-Rs (11, 121). Through CB1_R activation, cannabinoids have been shown to decrease cardiac contractility, possibly leading to heart failure (53–123). Although several mechanisms are implicated in cannabis-induced heart failure, clinical evidence is still inconsistent, and additional studies are needed.

### 3.6 Cannabis interaction with anticoagulants and antiplatelet agents

Anticoagulant and antiplatelet medications are commonly prescribed to high-risk patients with cardiac conditions as prophylaxis for myocardial infarction or stroke (124). Cannabis can be used for recreational or medicinal purposes in combination with commonly used anticoagulants and antiplatelet agents (125). These drug-drug interactions can lead to decreased efficacy of the medications and increase the risk of recurrent adverse cardiovascular and cerebrovascular events (126). This interplay is mediated by the inhibition of several CYP450 enzymes by cannabinoids (125, 126). One of the most studied relationships is between cannabis and warfarin, which can be separated into two stereoisomers, S-warfarin and R-warfarin (127–131). CYP2C9, CYP2C19, and CPY3A are the enzymes responsible for the breakdown of S-warfarin, while R-warfarin is metabolized by CYP1A2 and CYP3A (46); all these enzymes are moderately inhibited by the three major cannabinoids (THC, CBD, and CBN) (49, 127, 130, 131). CBD is a potent inhibitor of all the CYP enzymes involved in the metabolism of warfarin and can impact the degradation of warfarin and lead to higher plasmatic concentrations than needed (131, 132). R-warfarin, the least potent stereoisomer, is metabolized mainly by CYP3A4 (127, 132). This enzyme is involved in the metabolism of THC and CBD, as shown in clinical trial interaction studies of an oromucosal spray with *Cannabis sativa* extract (Sativex® U.K. SPC 7/2012). Several case studies have reported an increased international normalized ratio (INR) in cannabis users, which can place patients at risk for epistaxis, bruising, or intracranial hemorrhage (45, 49, 127, 128, 130). Clopidogrel is a popular antiplatelet agent that is used for the treatment of acute coronary syndrome, the prevention of heart attack, and cerebrovascular accidents (47, 133). The metabolism of clopidogrel by the isoenzyme CYP2C19 is inhibited by CBD, which could lead to lower levels of the active metabolite and increase the risk for transient ischemic attacks (47, 131, 134). There is no evidence of interactions between cannabis products and heparin or fondaparinux because they are cleared by the kidneys and have no apparent interactions with CYP enzymes (126, 131, 134).

Direct oral anticoagulants (DOACs) include Factor Xa inhibitors (rivaroxaban, apixaban, and edoxaban) and thrombin inhibitors (dabigatran) (135). DOACs have the potential to replace warfarin as the anticoagulant of choice due to their effectiveness and safety profile (136). DOACs act as substrates of the P-glycoprotein efflux system, which expels substances from cells to favor their metabolization (137). Several researchers have shown that prolonged exposure to cannabis can reduce the expression of P-pg and that CBD may be responsible for these effects (50, 137). Since P-glycoproteins are involved in the absorption of DOACs, the inhibitory effect of CBD on P-glycoproteins interferes with DOAC elimination and increases the plasma concentrations of the drug, increasing the risk of bleeding (50, 131). Additionally, other interaction mechanisms, such as rivaroxaban and apixaban, which are metabolized by the CYP450 enzyme system, similar to CBD, have been proposed for some DOACs. Therefore, the presence of CBD may alter the metabolism of these anticoagulants (138).

The anticoagulant effects of cannabis, as well as interactions with other drugs, are still being studied. In a study on rat models, THC and CBN inhibited thrombin activity and displayed anticoagulant activity (139). More clinical studies on the pharmacological interactions of cannabis in relevant patient populations are needed to understand the relative risk when using anticoagulants and antiplatelet medications (134).

### 3.7 Cerebrovascular outcomes

#### 3.7.1 Acute cerebral ischemia

The most common adverse cerebrovascular outcomes associated with cannabis and synthetic cannabinoids are acute cerebral ischemia and transient ischemic attacks, particularly in young individuals. (18–47 years) (140–142) Most case series and case reports show that more than 50% of ischemic strokes involve the anterior circulation, but they can also occur in the posterior circulation (143, 144). A case series showed that in 81% of ischemic stroke patients, cannabis consumption was related to the duration of the event; moreover, 22% of patients experienced recurrent cerebral ischemia after re-exposure to cannabis (143). A longitudinal study by Hemachandra et al. reported a 4.7-fold greater risk of cerebral ischemia in chronic cannabis users than in healthy individuals (145). Recently, Parekh et al. conducted a retrospective cohort study with data from the Behavioral Risk Factor Surveillance System Survey Analysis (2016–2017) and reported that recent cannabis use in young subjects (18–44 years of age) was associated with a 1.82 odds ratio (OR) of ischemic stroke compared with that in non-cannabis users; however, for heavy and frequent users, the risk of stroke increased 2.45-fold, suggesting a dose-effect relationship (146).

Multiple mechanisms have been proposed to explain the occurrence of cannabis-induced cerebral ischemia. Two prospective studies showed that young individuals presented with ischemic stroke and positive cannabis urine test results. angiographic studies revealed multiple intracranial vascular spasms, which reverted in some patients with total suppression of cannabis use (140, 147). Early preclinical studies in rats have shown a vasoconstrictor effect of THC (148). Moreover, Herning et al. conducted a prospective cohort study in which cerebral blood flow velocity was measured via transcranial Doppler (TCD) in 46 cannabis users (light, moderate, and heavy) and controls (noncannabis users) within three days of admission and up to 30 days of monitored abstinence. Their findings showed that cannabis users had significantly greater indexes of cerebrovascular resistance (>in heavy users) than did control subjects, which persisted after a month of monitored abstinence, suggesting a long-lasting cerebral vasoconstrictor effect of cannabis (149). The authors suggested that changes in cerebrovascular resistance with sustained cannabis use might be explained by the increased density of CB1-Rs in the brain vessels. Other studies in rat models have demonstrated that THC provokes dose-dependent impairment of mitochondrial function with the subsequent production of ROS and hydrogen peroxide, which leads to endothelial dysfunction in the cerebral vasculature (141). Other contributing factors are myocardial infarction and atrial fibrillation. Filbey et al. conducted a prospective study of 74 abstinent cannabis users for more than 72 h and 101 nonusers, aiming to compare intergroup differences in several neurophysiologic indicators, such as global and regional resting cerebral blood flow (CBF), oxygen extraction, and cerebral metabolic rate of oxygen (CMRO_2_). They reported that cannabis users had higher oxygen extraction and CMRO^2^ values as well as greater global and resting CBF values that correlated with THC levels. These findings demonstrated the residual effects of prolonged cannabis use (150).

#### 3.7.2 Cerebral hemorrhage

There is expanding evidence linking prolonged cannabis exposure to aneurysmal subarachnoid hemorrhage (aSAH) (151) and delayed ischemic stroke (152, 153); however, the pathophysiology of this association remains unclear. Nonetheless, some of the aforementioned neurophysiological events may contribute to multifocal intracranial stenosis, increased oxidative stress, and cerebral mitochondrial dysfunction. Recent retrospective cohort studies from secondary databases have shown a significantly greater incidence of hemorrhagic stroke in cannabis users than in nonusers (154, 155). In a retrospective study of consecutive patients admitted with aneurysmal subarachnoid hemorrhage, the neurologic outcomes were compared between patients with cannabis-positive urine test results and patients with negative drug screening results. Delayed cerebral ischemia was diagnosed in 50% of patients with CB-positive urine tests and 23.8% of those with negative drug screens (*P *= 0.01), and the incidence of poor neurologic outcome was greater in cannabis users (35.7% vs. 13.8%; *P *= 0.01) (151).

### 3.8 Pharmacological interactions of cannabinoids with anesthetic drugs

Pharmacological interactions of cannabis and cannabinoids with anesthetic drugs can lead to perioperative cardiovascular complications, although there is a paucity of clinical research studies showing robust evidence regarding the mechanisms involved in these interactions in humans. Most of the current knowledge on these interactions comes from preclinical studies in animal models (156–165), small clinical trials, and case reports (166–171). Interestingly, most of the described effects of cannabinoids and anesthetic agents, intravenous hypnotics, and opiates on animal models are the opposite of what has been described in case reports and clinical studies.

In clinical practice, the most prevalent outcome of these interactions is an increase in the need for inhalation and GABAergic intravenous anesthetics (propofol, thiopental, and benzodiazepines), manifested by resistance to achieve and maintain an adequate level of clinical anesthesia (monitored by the EEG bispectral index) (168, 170, 172–175). Cannabinoids interact with several receptors that are intimately involved in the mechanisms of general anesthesia, such as γ-amino-butyric acid (GABA), N-methyl-D-aspartate (NMDA), and vanilloid receptor 1 (TRPV1) (176). The interaction of THC, other cannabinoid compounds, and endocannabinoid ligands with GABA-A receptors leads to the formation of a CB1/GABA-A complex, impeding neurotransmission in GABAergic neurons and reducing the efficacy of the GABA-A receptor (126, 177, 178). Consequently, this interaction reduces the efficacy of GABAergic anesthetics (inhalation anesthetics and intravenous hypnotics). Additionally, upregulation of the hepatic cytochrome microsomal P-450 (CYP) enzymatic system occurs in heavy cannabis users, causing an increase in the expression and activity of the CYP system, which subsequently accelerates the metabolic degradation of propofol (179). These two pharmacological interactions affect the pharmacodynamic effects of inhalation and intravenous anesthetics, causing an increase in the required doses of these drugs to achieve sufficient drug levels in plasma and at the effector site (brain) to ensure adequate anesthesia depth (166, 169, 174, 180, 181) (Table 3).

**Table 3:** Potential pharmacological interactions of cannabis and cannabinoids with anesthetic agents.

Anesthetic and sedative drugs	Pharmacological interactions
Inhalational anesthetics and intravenous124, hypnotics (propofol, sodium thiopental, benzodiazepines)	• CB promote formation of CB1-R/GABA-A-R complex → ↓ efficacy of GABA-A-R and interferes with GABAergic neurotransmission → Increase inhalation and intravenous anesthetic requirements (51, 163, 164) • CB1_R activation by preoperative CB exposure → ↓ release of acetylcholine in neocortex → transient postoperative cognitive deficit • CB provokes changes in CYP enzymatic system expression and activity → acceleration in metabolic degradation of propofol and reducing its hypnotic effects (154, 156, 158–161)

CB, cannabis and/or cannabinoid; CB1-1, cannabinoid-1 receptor; GABA-A-R, γ-aminobutyric acid–a-receptor; THC, tetrahydrocannabinol; CYP, cytochrome P-450 enzymatic system.

### 3.9 Preoperative considerations of Cannabis users

Most of the evidence related to perioperative cardiovascular complications in cannabis users comes from case reports and small studies showing a low incidence of serious adverse events (181, 182). Nonetheless, recent evidence from retrospective studies from large national healthcare databases contradicts the findings of previous studies and highlights the importance of obtaining accurate information about cannabis use during preoperative assessments and discussing the potential risks of perioperative complications (183–187). In addition to cardiovascular complications, potential airway, and respiratory adverse outcomes can occur perioperatively in heavy users, such as upper airway obstruction by rhinopharyngitis and uveal edema, hyperreactive airways, high levels of carboxyhemoglobin, necrotizing alveolitis (188, 189).

During the preanesthetic evaluation, it is essential to determine whether the patient was a recent or chronic user if cannabis was used for medical or recreational purposes if the dose was most recently consumed if the time elapsed since the most recent exposure if the route or method of administration was used, if the type of cannabis or cannabinoid used was used, or if the THC: CBD ratio was used; if possible (190, 191) (Table 4). If the patient reports recreational use, the anesthesiologists should inquire about the use of the dried product, which usually contains a very heterogeneous mixture of its components, especially THC, whose concentration has increased progressively over time (192). It is also important to obtain information about the use of synthetic cannabinoids such as “K2” or “spice”. If the patient is taking medical formulations, it is imperative to know which product and dosage are in use; however, some cannabis products for medical use are marketed in an unregulated manner and may be labeled inaccurately (193). The combination of cannabis with other drugs, such as amphetamines and paroxetine, is becoming common, but usually, its presence may not be known to the user. A past medical history of hyperemesis episodes or severe “shivering” during a previous surgery should alert patients to the chronic use of cannabis (188).

**Table 4:** Preoperative evaluation of cannabis use.

1. Determine characteristics of consumption (173, 176, 177) • Chronic/heavy user vs. new user (176, 180) • Time since last consumption (176, 180) • Recreational vs. medical use (dose) (176, 180) • Type of recreational cannabis used (a) Dried cannabis has higher THC content (>20% THC/≥ 10 mg/serving) (177) (b) Use of synthetic cannabinoids (Spice, K2) (68) (c) Medical cannabinoids (composition, dose) (176, 180) 2. In heavy users (daily use or more than 6 times per week) • Inquire for past surgical history of: (150) • Episodes of hyperemesis • Heavy shivering after previous surgery (PACU) • Postoperative withdrawal syndrome (CWS) 3. Chronic cannabis users should be screened for Cannabis Use Disorder (176) 4. If possible, patients should start monitored weaning 5 days to 72 h before scheduled elective surgery (174, 176, 180) • Recent high THC dose consumption (>50 mg THC/serving) → delay elective surgery at least 2 h (174, 176, 179, 180, 183)^,^ • Inability to provide consent (up to 5 h) (180) • ↑risk MI with 2 h of smoking (180) • ↑risk of respiratory complications (174, 180, 181)

Recently, a consensus of a panel of experts from the Perioperative Pain and Addiction Interdisciplinary Network (PAIN) (194), as well as the ASRA Guidelines for Perioperative Management of Cannabinoid Users (190), made several recommendations related to the perioperative management of cannabis and cannabinoid users. Current evidence remains uncertain regarding the weaning period of recreational cannabis consumption, particularly in heavy users. Early studies on cannabis users undergoing general anesthesia and recent reviews suggested avoiding anesthesia in any patient who has used cannabis in the last 72 h (188, 189, 195, 196). ASRA guidelines recommend that “all surgical and procedural patients requiring anesthesia should be screened for cannabinoids preoperatively”, and patients who are referred for daily use of cannabis should be screened for cannabinoid use disorder (190, 194). If cannabis use is confirmed, patients must be evaluated clinically for acute intoxication. Drug testing for cannabis especially in urine, saliva, and hair detects evidence of use, not current intoxication or addiction, while blood samples allow to measure quantitative levels of cannabinoids (190, 197). Anesthesiologists and perioperative physicians should discourage the preoperative use of cannabis, except for medical indications. Recent or chronic heavy cannabis consumption poses a high risk of MI within two hours of smoking but also impairs cognitive functions and performance, including memory, and compromises the ability to provide informed consent for up to 5 h (198). ASRA guidelines recommend postponing elective surgery for at least 2 h for patients who exhibit symptoms of acute cannabis intoxication (190). According to the panel of experts, the consumption of 1.5 g/day of smoked cannabis, 300 mg/day of CBD oil, or 20 mg/day of THC oil should be considered significant. If assessed more than 7 days before surgery, monitored weaning should be considered; however, cannabis should not be stopped abruptly if the elapsed time for surgery is more than 24 h to avoid postoperative cannabis withdrawal syndrome (CWS) (190, 194).

### 3.10 Potential intraoperative cardiovascular adverse events in Cannabis users

An increase in the dose of anesthetics can lead to severe hypotension due to vasodilation and myocardial depression produced by agents such as propofol, isoflurane, sevoflurane, and desflurane (199–201).

New or naïve users are prone to tachycardia, systolic hypertension, and malignant arrhythmias (AFib, VFib, VTach, and Brugada pattern) within 2 h of consumption, depending on the dose administered due to sympathetic activation and parasympathetic inhibition (87, 202, 203). This sympathetic hyperactivity can potentiate the cardiovascular response to surgical stress, enhancing the increase in heart rate and contractility that some anesthetics, such as ketamine and other cardioactive drugs used intraoperatively (atropine, epinephrine), normally produce and, consequently, myocardial oxygen demand (95, 204). On the other hand, this sympathetic overactivity also counteracts the vasodilation and cardiac depression provoked by higher doses of GABAergic anesthetics.

Although the increase in heart rate following marijuana consumption ranges between 20% and 100% from baseline and reaches its maximum at 10–30 min after smoking, its high liposolubility may prolong tachycardia for up to 72 h (55). This initial response to inhaled cannabis could be followed by an increased parasympathetic tone characterized by bradycardia and hypotension (94, 202). The combination of high myocardial oxygen demands, coronary spasms, and elevated carboxyhemoglobin levels places patients at high risk for intraoperative myocardial infarction or malignant arrhythmias (Table 5) (205).

**Table 5:** Potential perioperative cardiovascular outcomes in cannabis users.

Intraoperative Period
1. New or naïve users (within 2 h of consumption) • Sympathetic hyperactivity + parasympathetic inhibition produced by THC potentiates cardiovascular response to surgical trauma and pain → ↑HR, ↑contractility, ↑MVO_2_ → Myocardial ischemia & malignant arrhythmias (68, 134, 135). 2. Increase anesthetic demands • Severe hypotension (vasodilation + myocardial depression) (130–132) • Sympathetic overactivity may offset hypotension induced by higher doses of GABAergic anesthetics. 3. Chronic and heavy users • Bradycardia and postural/orthostatic hypotension (30, 134). • Refractory hypotension when cannabis is combined with amphetamines (138).
Postoperative Period
• Myocardial Infarction (136, 163, 164, 167) • Arrhythmias (137) • Cerebrovascular ischemic events (163, 164, 167)^,^ • Deep venous thrombosis (165)

Conversely, chronic use of cannabis can produce bradycardia and postural and orthostatic hypotension; therefore, precautions should be taken with the use of neuraxial anesthesia, although no evidence of this interaction has been published to date (194). A recently reported case described an incident in which the combination of marijuana with amphetamines and paroxetine caused severe hypotension refractory to treatment with vasopressors such as phenylephrine shortly after the start of sevoflurane anesthesia (206).

### 3.11 Postoperative cardiovascular outcomes in cannabis users

Until recently, the reported incidence of postoperative cardiovascular events in cannabis consumers was considered very low or similar to that in noncannabis users; however, most of these data came from case reports and small-size prospective and retrospective studies (181, 182). In this subsection, we present findings from recent retrospective cohort-matched studies comparing the incidence of postoperative cardiovascular complications, such as myocardial infarction, arrhythmias, and ischemic cerebrovascular disease, in patients who used cannabis preoperatively with those who did not use cannabis (Table 5) (184, 185, 207–211).

A retrospective cohort study conducted by Goel et al. from a gross sample of 4,186,622 patients who underwent major elective surgery yielded a final cohort of 27,206 patients, with 13,603 patients in each group. Their results showed that chronic cannabis consumption was associated with an increased incidence of postoperative myocardial infarction [adjusted OR 1.88 (95% CI, 1.31–2.69; *P* < 0.001)] (*P* < 0.001]) (184). Chiu et al. conducted a study on 2,393 cannabis-using patients from a total of 423,978 patients who underwent elective spine surgery. Cannabis use was associated with a greater frequency of thromboembolic events (OR: 2.2; 95% CI 1.2–4.0; *P* = 0.005) and ischemic stroke (OR 2.9; 95% CI 1.2–7.5; *P* = 0.007) and an increased length of hospitalization compared to the control group (211). Zhang et al. studied 524 cannabis users who were undergoing surgery and compared them with a matched-pair cohort of 1,152 patients who were non-Cannabis users as controls. They found that cannabis users had a greater rate of arrhythmias than non-cannabis users (2.7% vs. 1.6%) (185). In another study, 3,842 cannabis users were identified from a total sample of 23,030 patients who underwent THA and were matched with 19,188 individuals in the control group. Cannabis users had greater postoperative cardiovascular complications, such as MI and CVA (23.0 vs. 9.8%, OR 1.6; *p *< 0.0001), and longer hospital stays than did the control group (208). A recent propensity score-matched cohort study of patients who underwent lumbar fusion from the secondary database (PearlDiver) reported that CBU patients had more myocardial infarction (*P* < .0001) and deep venous thrombosis (*P* < 0.0001) than did the control cohort (209). McGuinness et al., from a propensity score matching cohort of 4,684 patients with CUD from a total sample of 510,000 vascular surgery patients, reported that cannabis users had a greater incidence of perioperative MI (3.3% vs. 2.1%; *P* = .016) and perioperative stroke (5.5% vs. 3.5%; *P* = .0013) than patients without CUD (207).

## 4 Conclusion

The results of our search in the most recent publications on cardiovascular complications associated with the consumption of natural and synthetic cannabis show that its use may have important implications for the perioperative care of patients who are cannabis users and who require surgery under general anesthesia or sedation. Cannabis users, whether for recreational or medical purposes, may represent a challenge for the surgical team (anesthesiologists, surgeons, and hospitalists). In addition to the increase in THC concentration, the consumption of THC-containing products continues to increase, most noticeably among adolescents and young adults. The chronic and recent use of cannabis triggers a myriad of cardiovascular reactions that range from tachycardia, bradycardia, hypertension, and hypotension to malignant arrhythmias, myocardial infarction, and ischemic stroke throughout the entire perioperative period. However, the severity of the cardiovascular effects of cannabis may differ according to the duration of consumption and dose. Cannabis consumption also places patients at risk of pharmacological interactions with anesthetic agents, resulting in increased requirements for these drugs to maintain adequate levels of anesthesia. Cannabis also interacts with anticoagulants and antiplatelet drugs, modifying their efficacy.

This review highlights the potential for perioperative cardiovascular complications in surgical patients who consume cannabinoids. We acknowledge several weaknesses in this review due to several factors like (a) the literature relevant to this topic is of low quality as scientific evidence, especially because it is made up mostly of prospective and retrospective studies of small sample size, reports, or case series, which they generally do not clearly describe whether the adverse cardiovascular reaction is caused by the use of cannabis alone, without the influence of other drugs for recreational or medical purposes; (b) except for medical cannabis users, most patients with cannabis use disorder also consume other drugs that contribute to the appearance of adverse reactions mainly in the cardiovascular, respiratory, and nervous systems, therefore, knowing that the patient is an active, recent or chronic consumer, must alert the surgical team of the potential events that may occur acutely during the perioperative period. On the other hand, despite the legalization of the use of cannabis in 24 states in the United States and in other countries, the frequency with which the user manifests itself as such remains very low as shown by most studies (4%–5%), including recent epidemiological studies using repositories that contain data from millions of patients such as INS, Medicare/Medicaid, MarketScan, Cosmos-Epic, etc.

Despite the continued increase in the use of cannabis, both recreationally and medically, there is still high underreporting in our medical system of cannabinoid-consuming patients since the percentage of patients who report being consumers is still very low. Therefore, there is a need for additional large-size, prospective, and observational cohort clinical trials to characterize the perioperative and long-term consequences of cannabis use and the impact of preexisting comorbidities more accurately. The results of these studies will provide a wider knowledge to anesthesiologists and surgeons to more clearly identify the subset of patients most likely to experience severe postoperative adverse events.

## Data Availability

The original contributions presented in the study are included in the article/supplementary material, further inquiries can be directed to the corresponding author.
